# Effects of vatinoxan on gastrointestinal motility, sedation, and antinociception during and after long‐lasting detomidine infusion in horses

**DOI:** 10.1111/evj.14499

**Published:** 2025-03-20

**Authors:** Bartlomiej Obrochta, Heidi Tapio, Marja Raekallio, Luis Alfonso Gracia Calvo, Rebecca Rivera Pöyhönen, Kati Hagman, Noora Jantunen, Ninja Karikoski

**Affiliations:** ^1^ Faculty of Veterinary Medicine, Department of Equine and Small Animal Medicine University of Helsinki Helsinki Finland; ^2^ Veterinary Teaching Hospital University of Helsinki Helsinki Finland; ^3^ Faculty of Veterinary Medicine Estonian University of Life Sciences Tartu Estonia; ^4^ Royal (Dick) School of Veterinary Studies University of Edinburgh Midlothian UK

**Keywords:** alpha‐2 agonist, alpha‐2 antagonist, borborygmi, faeces, gastrointestinal transit, horse

## Abstract

**Background:**

Sedation in horses is typically achieved using alpha‐2 adrenoceptor agonists, although their use is associated with multiple side effects. A peripheral alpha‐2 adrenoceptor antagonist, vatinoxan, can alleviate many of these.

**Objectives:**

To evaluate the effects of vatinoxan infusion on gastrointestinal motility, sedation, and antinociception in horses sedated with detomidine infusion.

**Study Design:**

Randomised, blinded cross‐over in vivo experiments.

**Methods:**

Eight horses were given two 4‐h infusions: detomidine (0.01 mg/kg + 0.015 mg/kg/h IV) (DET) and a combination of detomidine and vatinoxan (0.15 mg/kg + 0.05 mg/kg/h IV) (DET + VAT). Plastic marker balls were administered via nasogastric entubation before the start of the infusion. Borborygmi score was monitored. The expelling of balls and faecal output were repeatedly monitored for 72 h after the infusion. Sedation score (SS) and antinociception were monitored during the infusion.

**Results:**

Borborygmi score remained significantly higher during DET + VAT infusion and the following hour than with DET (*p* < 0.05) at different time points. Median (range) cumulative weight of faeces was significantly higher with DET + VAT [6.25 kg (3.52–8.65)] than with DET [2.85 kg (1.7–6.6)] (*p* = 0.007) during the first 8 h after the end of infusion. The markers were expelled significantly faster after DET (mean AUC_12–72_ 3928 ± 1620) than after DET + VAT (mean AUC_12–72_ 2460 ± 1199) infusion (*p* = 0.02). All animals were appropriately sedated. Median SS was significantly lower at 60 min during DET + VAT [7 (5–7)] than during DET [7.5 (7, 8)] (*p* = 0.04). No other significant differences were detected in SS or antinociception between treatments.

**Conclusions:**

Vatinoxan significantly improved the borborygmi score. Horses treated with DET + VAT passed more faeces in the initial period after infusion, although the expelling of plastic balls was faster with DET. Combining vatinoxan with detomidine infusion may slightly reduce the level of sedation in the initial phase of infusion. Vatinoxan may improve gastrointestinal motility in horses treated with detomidine infusion.

## Resumo

**Contexto:**

Agonistas dos receptores adrenérgicos alfa‐2 são comumente utilizados para sedação em cavalos, embora seu uso esteja associado a múltiplos efeitos colaterais. Um antagonista periférico do receptor adrenérgico alfa‐2, vatinoxan, pode aliviar muitos desses efeitos.

**Objetivos:**

Avaliar os efeitos da infusão de vatinoxan na motilidade gastrointestinal, sedação e antinocicepção em cavalos sedados com infusão de detomidina.

**Desenho do Estudo:**

Estudo in vivo, randomizado, cego e cruzado.

**Métodos:**

Oito cavalos receberam duas infusões de quatro horas: detomidina (0,01 mg/kg + 0,015 mg/kg/h IV) (DET) e uma combinação de detomidina e vatinoxan (0,15 mg/kg + 0,05 mg/kg/h IV) (DET + VAT). Bolas marcadoras de plástico foram administrada via tubo nasogástrico antes do início da infusão. O escore de borborigmos foi monitorado. A expulsão das bolas e a produção faecal foram monitoradas repetidamente por 72 horas após a infusão. O escore de sedação (ES) e a antinocicepção foram monitorados durante a infusão.

**Resultados:**

O escore de borborigmos permaneceu significativamente mais alto durante a infusão de DET + VAT e na hora seguinte do que com DET (*p* < 0,05) em diferentes momentos. O peso cumulativo mediano (intervalo) das fezes foi significativamente maior com DET + VAT [6,25 kg (3,52‐8,65)] do que com DET [2,85 kg (1, 6)] (*p* = 0,007) nas primeiras 8 horas após o fim da infusão. Os marcadores foram expelidos significativamente mais rápido após DET (média AUC_12‐72_ 3928 ± 1620) do que após a infusão de DET + VAT (média AUC_12‐72_ 2460 ± 1199; *p* = 0,02). Todos os animais foram adequadamente sedados. O ES mediano foi significativamente menor aos 60 minutos durante DET + VAT [7 (5–7)] do que durante DET [7,5 (7, 8)] (p = 0,041). Nenhuma outra diferença foi detectada no ES ou na antinocicepção entre os tratamentos.

**Conclusões:**

Vatinoxan melhorou significativamente o escore de borborigmos. Cavalos tratados com DET + VAT passaram mais fezes no período inicial após a infusão, embora a expulsão de bolas de plástico tenha sido mais rápida com DET. A combinação de vatinoxan com a infusão de detomidina pode reduzir ligeiramente o nível de sedação na fase inicial da infusão. Vatinoxan talvez melore a motilidade gastrointestinal em cavalos tratados com infusão de detomidina.

## 1 INTRODUCTION

Despite considerable progress in the field of veterinary anaesthesia, the risk associated with surgical and diagnostic procedures performed under general anaesthesia is relatively high in horses.^1^, ^2^ An increasing number of veterinary procedures are currently performed on standing horses to reduce this risk.^2^, ^3^ Sedation is typically achieved with the use of boluses or constant rate infusion (CRI) of alpha‐2 adrenoceptor agonists (alpha‐2 agonists),^2^ as they have been shown to provide reliable levels of sedation and antinociception (inhibition of the detection of a painful stimulus by sensory neurons).^4^, ^5^ However, alpha‐2 agonists also cause side effects such as reduced gastrointestinal motility^6^, ^7^, ^8^, ^9^, ^10^ increased urine production^9^ peripheral vasoconstriction leading to hypertension and reflective bradycardia,^11^ and disturbances in glucose metabolism.^12^, ^13^ Long‐lasting infusions of alpha‐2 agonists are suspected to increase the risks of gastrointestinal disturbances, such as impaction colic, based on their influence on gastrointestinal motility,^6^, ^7^, ^8^, ^9^, ^10^, ^14^ but studies on this topic are lacking in horses undergoing standing sedation.

Vatinoxan is an alpha‐2 adrenoceptor antagonist that mainly inhibits peripheral adrenergic receptors because it only minimally crosses the blood–brain barrier.^15^, ^16^ Vatinoxan has been shown to improve gastrointestinal motility and faecal output in horses receiving alpha‐2 agonists.^17^, ^18^, ^19^ It also alleviates the impact of alpha‐2 agonists on cardiovascular functions^17^, ^18^, ^19^ and glucose metabolism.^13^ Although vatinoxan does not markedly affect the level of sedation by central mechanisms, it may decrease the plasma concentration of the alpha‐2 agonist via improved haemodynamics.^17^, ^18^, ^20^ The influence of vatinoxan on alpha‐2 agonist‐induced antinociception or faecal transit time has not been evaluated in horses earlier.

The aim of this study was to investigate the effects of vatinoxan CRI on gastrointestinal motility, sedation, and antinociception in horses administered a 4‐h detomidine CRI. We hypothesised that vatinoxan would improve gastrointestinal motility, it would not markedly affect the sedation and antinociception produced by detomidine, and it would decrease the concentration of detomidine in plasma during CRI.

## 2 MATERIALS AND METHODS

### 2.1 Animals

Eight adult Finnhorses owned by the Natural Resources Institute Finland and Harju Vocational College were included in this study. The median age of the horses was 13 years (range 4–16 years). There were six mares and two geldings, and their median weight was 587 kg (range 550–620 kg). Until the start of the study, all horses were kept in a loose housing system where they had access to dry hay for 160–250 min/day (median 200 min, divided evenly throughout the day) and free access to straw. Additionally, horses received oat grains 50–1000 g/day (median 100 g) and minerals. Ad libitum fresh water was provided throughout the day. All horses were considered healthy based on clinical examination and basic haematology and serum biochemistry results before each treatment.

### 2.2 Study design

The study design was an experimental, randomised, blinded cross‐over study.

### 2.3 Test procedures

In the morning of each treatment day, horses were restrained in stocks. Both jugular veins were aseptically prepared and 14G intravenous catheters placed (Intraflon, Génia). One catheter was used for drug administration and the other for blood sampling. Each horse was assigned to two treatments by a randomised (coin toss) cross‐over study design with 21–27 days (median 21 days) between treatments: (1) detomidine hydrochloride (HCl) (Equisedan 10 mg/mL, Vetcare Ltd.) loading dose 0.01 mg/kg IV, followed by CRI 0.015 mg/kg/h (DET) and (2) the same doses of detomidine HCl combined with vatinoxan HCl powder (Vetcare Ltd.). The powder was dissolved in saline to a concentration of 10 mg/mL before preparing the loading dose and CRI. The loading dose of vatinoxan was 0.15 mg/kg IV, and this was followed by CRI at 0.05 mg/kg/h (DET + VAT). The loading dose was administered 5 min before the beginning of CRI (T0), which was administered using an infusion pump (B. Braun, Melsungen, Germany) and continued for 4 h. The CRI was prepared by adding both medications into a 1 L bag of sterile saline. After the end of the infusion, horses were continuously observed for four more hours after which the intravenous catheters were removed.

### 2.4 Gastrointestinal tract function

#### 2.4.1 Faecal output and gastrointestinal passage time

Faecal collection was conducted during the following 72 h after the end of CRI. Faeces were collected every 4 h from the stall, placed in closed plastic bags, and weighed on a calibrated scale.

For evaluation of gastrointestinal passage time, 200 coloured plastic marker balls (6 mm diameter, weight 0.12 g) were administered for each animal via nasogastric tube and flushed with 3 L of tap water after the loading dose of DET or DET + VAT. The faecal material was manually searched for expelled plastic balls starting from faeces passed between 8 and 12 h after the end of CRI. Area under the cumulative number of expelled balls‐time (12–72 h) curve (AUC_12–72_) was calculated for both treatments using a trapezoidal method and analysed.

All horses were turned out of the loose housing system every day for 4 h (12 AM to 4 PM) to provide daily exercise. During this time, the faecal output was not monitored, and faeces were not collected.

#### 2.4.2 Faecal moisture

Samples for determining faecal moisture were taken at baseline before the loading dose and then every 8 h after the end of CRI over the following 72 h. Samples were collected into double plastic zip‐sealed bags and stored at −20°C. Twenty‐four hours before the analysis, samples were defrosted in a fridge at 5°C. Each sample was manually mixed to achieve adequate homogeneity, and 2‐g specimens were analysed (Moisture Analyzer MB23, OHAUS Europe GmbH). Three parallel specimens were analysed for the first half of the samples. The variation between the parallel analyses was minor; thus, a single analysis was performed for each of the remaining samples. For parallel analyses, the mean of three results was used for further data analyses.

#### 2.4.3 Borborygmi

Intestinal borborygmi were auscultated and scored according to a previously defined system (Scoresheet S1)^9^ before sedation, thereafter at T30, T60, T120, T180, and T240, and then every 60 min for 4 h after the end of CRI. The horses were auscultated by the researchers unaware of the treatment.

### 2.5 Antinociception

Antinociception was recorded using a hand‐held pressure algometer (Prod, Topcat Ltd.). Hair from the dorsal aspect of left front limbs was clipped at the level of the mid‐cannon bone (5 × 5 cm area). The 2‐mm flat‐ended probe was pressed against the limb in the middle of the clipped area and pressure was gradually increased. At the point when the horse reacted (lifted the limb), the pressure was discontinued immediately, and the value was recorded. Baseline measurements were obtained ~5 min before the administration of the loading dose, after the horses had settled down in stocks. Measurements were obtained at time points T30, T60, T120, T240, and at 60 min after the end of CRI by researchers unaware of the treatment. 

### 2.6 Level of sedation

The level of sedation was scored according to a previously defined scoring system (Scoresheet S2)^21^ by researchers unaware of the treatment at baseline, at time points T30, T60, T120, T180, and T240, and at 60 min after the end of CRI. In the score, zero was totally awake and 10 was a very deeply sedated but still standing horse.

### 2.7 Heart rate

The heart rate was recorded by 30 s auscultation and multiplied by two by researchers unaware of treatment at baseline, at time points T30, T90, T120, T180, and T240, and at 60 min after the end of CRI.

### 2.8 Plasma drug concentrations

Venous blood samples were taken into five‐millilitre K2 EDTA tubes (Vacuette, Greiner Bio‐One GmbH) at time points T60, T120, T180, and T240. Samples were taken from the separate intravenous catheter used exclusively for blood sampling and flushed afterwards with saline–heparin solution. The samples were centrifuged, and the plasma was stored at −20°C. The concentrations of detomidine and vatinoxan were analysed with high‐performance liquid chromatography–tandem mass spectrometry in separate aliquots that were processed under specific conditions for each analyte, as described earlier.^19^

Area under the plasma detomidine concentration‐time (60–240 min) curve (AUC_60–240_) was calculated for both treatments using a trapezoidal method. Steady state clearance (CL_SS_) was calculated with the formula CL_SS_ = D_CRI_/C_SS_, where D_CRI_ was the infusion rate and C_SS_ was the concentration of the drug in plasma at the end of CRI. The doses of detomidine and vatinoxan were used in the formula without HCl; thus, the dose of detomidine was 12.54 μg/kg/h and the dose of vatinoxan was 46 μg/kg/h.

### 2.9 Monitoring of adverse effects

Horses were continuously observed for signs of abdominal discomfort or other signs of illness during the CRI and for the following 4 h; thereafter, they were inspected every 4 h over the next 72 h. All horses were clinically examined twice a day in the 3 days following drug administration. Any signs of gastrointestinal discomfort, such as decreased appetite, teeth grinding, pawing, flank watching, repeated rolling, loose faeces, abdominal distention, or other signs of illness were recorded.

### 2.10 Data analysis

An online statistical calculator was used for sample size calculations (https://statulator.com/). For faecal output, a sample size of five horses was required to achieve a power of 80% and a level of significance of 5% for detecting a mean difference of 3 kg between treatments, assuming the standard deviation (SD) of the difference to be 1.5 kg. For detecting a mean difference of 10 N between treatments in the mechanical antinociception, with a 7 N SD of the differences, a minimum of seven horses was required. Therefore, eight horses were included.

All data collected during the study period were recorded, and a statistical analysis was performed using IBM SPSS Statistics version 29.0.0 (IBM, SPSS Inc.). The normality of the dataset was evaluated using the Shapiro–Wilk test. Normally distributed parametric data were processed using repeated measures ANOVA models and paired samples Student's *t*‐test. Non‐parametric data were evaluated with the Wilcoxon signed‐rank test. Results with a *p*‐value <0.05 were considered statistically significant.

## 3 RESULTS

### 3.1 Gastrointestinal tract function

**FIGURE 1:**
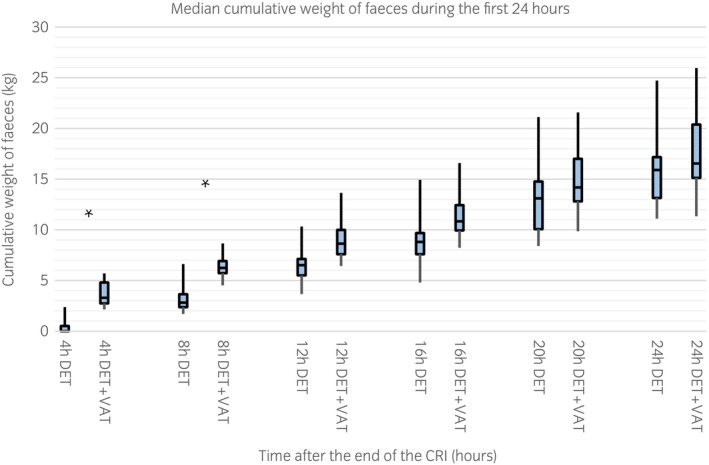
Median cumulative weight of faeces collected within the first 24 h after the end of detomidine (DET) or detomidine‐vatinoxan (DET + VAT) CRI in eight Finnhorses. Minimum and maximum values indicated by whiskers; horizontal bar represents median. Significant differences between the groups are marked with an asterisk (*p* < 0.05).

Median cumulative weight of faeces was significantly higher with DET + VAT than with DET during the first 8 h after the end of CRI (Figure 1). No significant differences were detected at later time points. Horses treated with DET + VAT passed higher numbers of faecal piles within the first 4 h after the end of CRI [DET median 0 (minimum 1–maximum 3); DET + VAT 2 (1–5)]; later, the difference between treatments was no longer observed.

**FIGURE 2:**
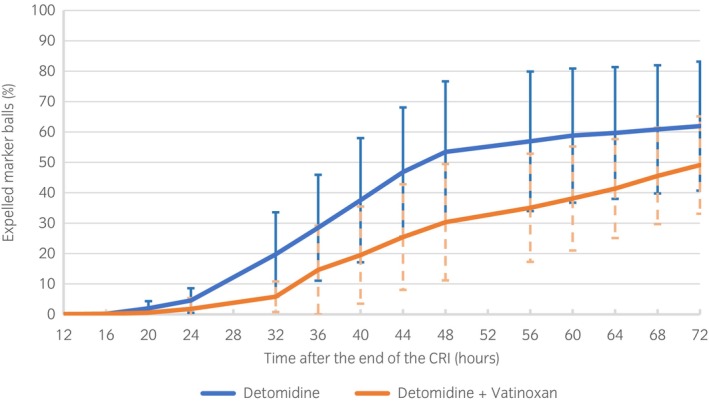
Cumulative proportion of expelled marker balls (mean ± SD) in faeces presented as the percentage of the total number of marker balls administered to eight Finnhorses during the 72‐h monitoring period after the end of detomidine (DET) or detomidine–vatinoxan (DET + VAT) CRI.

Area under the cumulative number of expelled balls‐time (12–72 h) curve (AUC_12–72_) was significantly higher after DET than after DET + VAT infusion (*p* = 0.02). Horses treated with DET expelled a higher number of balls in a shorter period than horses treated with DET + VAT (Figure 2).

Faecal moisture ranged from 73.5% to 86.0%. No significant differences were detected over time or between treatments. None of the horses developed watery faeces over the monitoring period. Borborygmi score remained significantly higher during DET + VAT CRI and the following hour than during DET (Table 1). The borborygmi score returned to baseline within 60 min after the end of DET + VAT (*p* = 0.09), whereas with DET the score remained significantly lower for the whole 4‐h monitoring period (*p* < 0.05).

**TABLE 1:** Borborygmi score [median (minimum‐maximum)] during and after the end of detomidine (DET) or detomidine–vatinoxan (DET + VAT) CRI in eight Finnhorses.

	Before CRI	30 min	60 min	120 min	180 min	240 min	300 min	360 min	420 min	480 min
Borborygmi DET	5.75 (2.5–8)	0.25 (0–2)	0.5 (0–2)	0.25 (0–2)	0.25 (0–2)	0 (0–2.5)	1 (0–3.5)	1.75 (0–6)	2.5 (0.5–8)	2.5 (0–5)
Borborygmi DET + VAT	4.5 (3–11)	3.5 (1.5–9)	3.25 (1.5–8)	2 (0.5–9)	1.5 (0–9)	2.75 (0.5–9)	4.5 (1–6)	4.25 (1.5–10)	4.5 (3–10)	5 (2–12)
*p*‐value	0.9	0.02*	0.02*	0.04*	0.09	0.04*	0.03*	0.09	0.1	0.07

*Note*: Significant differences between the treatments are marked with an asterisk (*p* < 0.05).

### 3.2 Sedation score

All horses were visibly sedated during both CRIs. At T60, but not at the other time points, the sedation score was significantly higher with DET than with DET + VAT (Table 2).

**TABLE 2:** Sedation scores [median (minimum–maximum)], algometer readings (*N*) (mean ± standard deviation), heart rates (beats/min) [median (minimum–maximum)], and concentrations of detomidine and vatinoxan in plasma (ng/mL) (mean ± standard deviation) in eight Finnhorses treated with detomidine (DET) and detomidine–vatinoxan (DET + VAT) CRI for 4 h.

Treatment	Before	30 min	60 min	120 min	180 min	240 min	300 min
Sedation score							
DET	0.5 (0–2)	8 (6–8)	7.5 (7–8)	7 (7–7)	7 (7–7)	7 (6–7)	2 (1–5)
DET + VAT	1 (0–1)	7 (6–7)	7 (5–7)*	7 (6–7)	7 (6–7)	7 (6–7)	1 (0–6)
Algometer (*N*)							
DET	9.8 ± 6.6	20.7 ± 4.5	22.9 ± 3.4	20.3 ± 5.8	N/A	20.5 ± 2.4	15.3 ± 5.5
DET + VAT	8.6 ± 7.2	17.3 ± 4.8	20.0 ± 5.2	18.8 ± 6.5	N/A	21.1 ± 3.1	13.5 ± 6.7
Heart rate (beats/min)							
DET	38 (28–46)	25 (20–32)	25 (20–30)	24 (20–30)	24 (20–34)	24 (18–34)	32 (26–40)
DET + VAT	36 (24–62)	32 (28–36)*	32 (26–34)*	30 (26–32)*	29 (24–30)	26 (24–30)	33 (24–42)
Plasma concentration of detomidine (ng/mL)							
DET	N/A	N/A	8.2 ± 3.6	8.8 ± 3.8	11.4 ± 2.3	11.4 ± 4.3	N/A
DET + VAT	N/A	N/A	6.1 ± 1.4	8.0 ± 2.1	8.0 ± 1.8	8.9 ± 2.6	N/A
Plasma concentration of vatinoxan (ng/mL)							
DET + VAT	N/A	N/A	100 ± 19	116 ± 36	113 ± 25	122 ± 40	N/A

*Note*: Significant differences between the treatments are marked with an asterisk (*p* < 0.05).

### 3.3 Antinociception

During both CRIs at all time points, the algometer readings were significantly higher than at baseline, and no differences were detected between the treatments (Table 2).

### 3.4 Plasma drug concentration

No significant differences were detected between treatments in plasma detomidine concentrations at any single time point (Table 2), but AUC_60–240_ for detomidine during DET + VAT was significantly lower than during DET (23.5 ± 3.8 vs. 30.0 ± 7.8 mL/min/kg; *p* = 0.03). Mean AUC_60–240_ for DET + VAT was 81.1 ± 18.3% of that for DET. CL_SS_ for detomidine was 21.5 ± 9.6 mL/min/kg in DET and 25.4 ± 8.1 mL/min/kg in DET + VAT (*p* = 0.4). CL_SS_ for vatinoxan was 6.8 ± 1.8 mL/min/kg.

### 3.5 Heart rate

Heart rate was significantly reduced from baseline during both infusions. It was significantly higher with DET + VAT than with DET during the first 120 min (Table 2).

### 3.6 Adverse effects

Two horses showed mild signs of abdominal discomfort (lying down in sternal position, poor appetite) within the first 4 h after the end of CRI with DET + VAT. One of these horses showed similar signs after DET alone. The horses were walked in hand, and the signs resolved spontaneously without any pharmacological treatment within the following 2 h.

## 4 DISCUSSION

Our results suggest that vatinoxan used together with detomidine in long‐lasting CRI may alleviate some negative effects of alpha‐2 agonists on gastrointestinal motility during the infusion. Meanwhile, it does not markedly impact the level of sedation or antinociception. Although horses treated with DET + VAT had higher borborygmi scores during the CRI and the first hour thereafter and passed more faeces within the first 8 h after the end of treatment, no difference was detected during the later phase of the study. However, horses treated with DET expelled the plastic balls significantly faster than horses with DET + VAT.

The influence of vatinoxan on detomidine‐induced gastrointestinal effects is controversial. Although alpha‐2 agonists have a negative impact on the motility of the, caecum, large colon, and proximal aspect of the small intestines,^6^, ^7^, ^8^, ^9^ gastrointestinal motility is a complex combination of synchronised propulsive and retrograde movements of the different parts of the gastrointestinal tract.^22^ These provide an adequate environment for mixing of the ingesta and passaging to the further segments of the gastrointestinal tract. Dysregulation in the balance between these processes can affect the gastrointestinal passage time. In equids, these processes are essential particularly in the caecum and large colon.^22^, ^23^, ^24^ Although vatinoxan significantly improved the borborygmi score, it may not be tantamount to maintaining the physiological balance between these processes during alpha‐2 agonist‐induced sedation.

The dysregulation of propulsive and retrograde gastrointestinal movements could help to explain the faster expelling of markers observed in horses sedated with detomidine. Markers mixed with water may have moved through the stomach without mixing with its contents and ended up in the caecum where the whole content should have been mixed and then moved into the large colon. The process of mixing normally continues in the large colon led by retrograde movements originating from the pelvic flexion area.^25^ Detomidine, by affecting this process, could have prevented adequate mixing of ingesta, resulting in faster passage of the soft or watery content with plastic markers. In addition, lowered contractility of the caecum wall could have enhanced the fast bypassing of the light watery content into the large colon. Both phenomena may at least in part explain the shorter gastrointestinal passage time for the plastic balls in horses treated with DET than in those treated with DET + VAT. However, a higher borborygmi score, number of faecal piles, and median cumulative weight of faeces indicated better gastrointestinal motility with DET + VAT. Thus, vatinoxan might have enhanced the physiological movements of the gastrointestinal tract in horses sedated with DET, as indicated by the higher borborygmi scores and passing more faeces. Maintaining the large intestine movements by vatinoxan would be beneficial since clinical data from horses that underwent standing sedation with alpha‐2 agonists showed that these animals may be under a greater risk of caecal impaction.^26^ However, the effects of alpha‐2 agonists and antagonists on the function of different parts of the equine gastrointestinal tract require further investigations preferably with placebo treatment.

Vatinoxan significantly decreased the AUC_60–240_ of detomidine plasma concentrations. A similar phenomenon has been reported earlier for various alpha‐2 agonists in many species after IV bolus injections.^16^, ^17^, ^18^, ^27^, ^28^, ^29^ This has been explained by increased CL of alpha‐2 agonists by vatinoxan due to improved perfusion of the elimination organs. However, in our study, CL_SS_ for detomidine was not significantly affected by vatinoxan. Differences in HR could no longer be detected in the latter half of the CRI, suggesting that the cardiovascular status, and thus, also organ perfusion might already have been quite similar in both treatments at the time when CL_SS_ was evaluated.

Despite the lower plasma concentration of detomidine during DET + VAT CRI than during DET CRI, no significant difference between treatments was detected in the antinociception induced by detomidine. This is in contrast to an earlier report, where medetomidine‐induced antinociception was significantly reduced by vatinoxan in dogs.^27^ In that study, the reduction of the alpha‐2 agonist concentration in plasma by vatinoxan was clearly larger than in our study. In another canine study with higher doses of medetomidine and vatinoxan, no significant difference was detected in the level of sedation and antinociception in spite of the marked reduction of medetomidine concentration in plasma by vatinoxan.^29^ In our study, a slight reduction by vatinoxan was only detected in the level of sedation at one time point. Our results agree with earlier reports in which the level of sedation was monitored in horses after bolus injections of detomidine with and without vatinoxan.^17^, ^19^ However, in our study, all horses showed good clinical sedation (visibly sedated without difficulty to stand) during the CRI, and thus, these minor differences detected between treatments were probably not clinically relevant.

### 4.1 Limitations

Plastic balls were chosen as markers for gastrointestinal transit time based on previous studies where they had been successfully used with a similar follow‐up period.^30^, ^31^ However, a high proportion of the plastic balls were not retrieved during our follow‐up. Due to practical reasons and animal welfare issues, horses were turned out together with others, where they could not be continuously monitored, and faeces were not collected for 4‐h periods on the days following the end of the infusion. These circumstances were similar for all animals and for both treatments, and thus, they should not bias the comparisons between treatments, although the exact total number of balls expelled during the follow‐up is not known. In addition, the balls settling in the caecum or large colon may have confounded the interpretation of results of gastrointestinal transit time, as suggested by another group who reported incomplete retrieval of video endoscopy capsules.^32^ In that study, a large proportion of markers were also lost, and wide individual variation was detected in the time that the last markers were expelled. The maximum time was as high as 43 days.^31^

## 5 CONCLUSIONS

Combining vatinoxan with detomidine in long‐lasting (4 h) infusions significantly improved borborygmi scores. Horses treated with DET + VAT passed more faeces in the initial period after the infusion, although the expulsion of marker balls was faster with DET. Therefore, vatinoxan may benefit gastrointestinal motility in horses treated with detomidine infusion. Vatinoxan significantly decreased the concentration of detomidine in plasma and the level of sedation, but not antinociception, in the initial period of the infusion; however, the difference was small and thus clinically irrelevant. Further research is needed to evaluate the impact of vatinoxan on gastrointestinal motility in horses.

## FUNDING INFORMATION

This study was funded by Vetcare Ltd., Finland.

## CONFLICT OF INTEREST STATEMENT

The authors have no conflicts of interest to declare.

## AUTHOR CONTRIBUTIONS

**Bartlomiej Obrochta:** Data curation; writing – original draft; methodology; writing – review and editing; investigation. **Heidi Tapio:** Conceptualization; writing – original draft; writing – review and editing; methodology; investigation. **Marja Raekallio:** Conceptualization; formal analysis; writing – original draft; methodology; investigation; supervision; writing – review and editing; funding acquisition. **Luis Alfonso Gracia Calvo:** Conceptualization; writing – review and editing; investigation. **Rebecca Rivera Pöyhönen:** Data curation; investigation. **Kati Hagman:** Data curation; investigation; methodology. **Noora Jantunen:** Data curation; investigation. **Ninja Karikoski:** Conceptualization; methodology; investigation; data curation; writing – original draft; supervision; writing – review and editing.

## DATA INTEGRITY STATEMENT

Bartlomiej Obrochta and Marja Raekallio had full access to all the data in the study and take responsibility for the integrity of the data and the accuracy of the data analysis.

## ETHICAL ANIMAL RESEARCH

The study protocol was approved by the National Animal Experimentation Board of Finland (Approval number: ESAVI/23464/2022; Approval date: 11.8.2022).

## INFORMED CONSENT

Not Applicable.

## ANTIMICROBIAL STEWARDSHIP POLICY

Not applicable.

## Supporting information


**Data S1.** Supporting Information.

**Data S2.** Supporting Information.

## Data Availability

The data that support the findings of this study are openly available in Zenodo at https://doi.org/10.5281/zenodo.15023835.
