# Supplementary figures and images for: Effects of vatinoxan on gastrointestinal motility, sedation, and antinociception during and after long‐lasting detomidine infusion in horses
Source: Equine Vet J. 2025 Mar 20;58(1):212–9. doi: 10.1111/evj.14499 (PMC12699100; doi:10.1111/evj.14499)

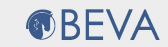[illegible]

Supplement: Supplementary file 1 — Data S1. Supporting Information. [file EVJ-58-212-s002.pdf]
