# Supplementary material for: Effects of vatinoxan on gastrointestinal motility, sedation, and antinociception during and after long‐lasting detomidine infusion in horses
Source: Equine Vet J. 2025 Mar 20;58(1):212–9. doi: 10.1111/evj.14499 (PMC12699100; doi:10.1111/evj.14499)
Supplement: Supplementary file 2 — Data S2. Supporting Information. [file EVJ-58-212-s001.pdf]

Equine Veterinary Journal BEVA

Scoresheet S2: Sedation scoring sheet.

Horse: ___________________________________ Date _______ / _______ 202__
Person who evaluated: _________________

SEDATION SCORE	Baseline	30 min	60 min	120 min	240 min	300 min	Comments
**General attitude**							
Nervous 0							
Calm 1							
Apathetic 2							
Stuporous 3							
**Standing ability**							
Stands well 0							
Leans slightly 1							
Leans strongly 2							
Difficulties in standing 3							
**Head**							
Moving 0							
Quiet 1							
Hanging on the halter 2							
**Eyes**							
Normal 0							
Slightly closed 1							
**Ears**							
Moving 0							
Not moving 1							
**Total sedation score**							
